# Salinity tolerance and desalination properties of a *Haematococcus lacustris* strain from eastern Hungary

**DOI:** 10.3389/fmicb.2024.1332642

**Published:** 2024-03-14

**Authors:** István Bácsi, Aida Figler, Edina Simon, Majd Muwafaq Yaqoob, Kamilla Márton, Viktória B-Béres

**Affiliations:** ^1^Department of Hydrobiology, University of Debrecen, Debrecen, Hungary; ^2^HUN-REN−UD Functional and Restoration Ecology Research Group, Debrecen, Hungary; ^3^Department of Bioinformatics, Semmelweis University, Budapest, Hungary; ^4^Department of Ecology, University of Debrecen, Debrecen, Hungary; ^5^HUN-REN–UD Anthropocene Ecology Research Group, Debrecen, Hungary; ^6^Functional Algology Research Group, HUN-REN–Centre for Ecological Research, Institute of Aquatic Ecology, Debrecen, Hungary

**Keywords:** salt-treatment, drying out, *Haematococcus*, ion removal, cyst formation, cyst maturation

## Abstract

Nowadays the increasing amount of saline wastewaters has given rise to various biological desalination processes, among which the application possibilities of microalgae represents a priority research area. Next to “real” aquatic species (members of phytoplankton or phytobenthon), species from ephemeral aquatic habitats or aeroterrestrial algae also could be good candidates of research studying salt tolerance or desalination ability, since salinity stress is often referred as “physiological drought” and species from ephemeral habitats can be characterized by high drought tolerance. In this study, the salinity tolerance, salt and nutrient removal ability of a *Haematococcus lacustris* strain from eastern Hungary were investigated. Vegetative cells showed low salt tolerance, survival was ensured by the formation of cysts up to a sodium-chloride concentration of 2,000 mg l^−1^. Although relatively moderate (a max. 30%) conductivity reduction and chloride removal were observed, notable (nearly 100%) nitrate and phosphate removal occurred even in the presence of 2,000 mg l^−1^ NaCl. Carotenoid accumulation was observed earlier and in higher extent in salt treated cultures than in drying out ones, although the amount of astaxanthin-esters was significantly higher in the cultures of drying out experiment than in the corresponding cultures of salt treatment characterized with similar chloride content. Our results suggest that algae isolates from ephemeral aquatic habitats endangered by regular drying out (exposed to special salt stress), could have notable salt tolerance and consequently successful applicability in nutrient removal processes from slightly saline wastewaters. The accumulation of valuable metabolites (such as astaxanthin) as a response to salinity stress, could enhance the economic value of the biomass.

## 1 Introduction

Salinization is one of the most important threat affecting freshwater ecosystems and endangering water resources (Reid et al., 2019; Ahmed et al., 2023). High number of agricultural and industrial activities, and even households contribute to the increasing amounts of saline wastewaters (Liang et al., 2017). The reduction of salt content of saline wastewaters is necessary before they could be introduced to general wastewater treatment systems or to surface waters. Relatively cheap methods, like dilution (e.g., addition of rainwater), or precipitation, coagulation/flocculation, sedimentation are used mainly as pretreatment technologies (Abushawish et al., 2023). These methods are rarely efficient enough alone as final purification methods, but usually are required before further physical, chemical or biological treatments. Physical and chemical treatments (distillation, vacuum evaporation, ion exchange, electrodialysis, reverse osmosis, etc.) generally have significant investment and operational costs (Ahmed et al., 2023) and their use may cause additional environmental problems (Shokri and Fard, 2023). Biological desalination processes increasingly appear to be the most favorable solutions: micro and macroorganisms both are studied and/or used for reducing water or soil salinity (Taheri et al., 2016).

Among microorganisms, beside bacteria and fungi, microalgae are known to be able to remove a wide range of pollutants from many types of wastewaters (Delrue et al., 2016) and some of them tolerate well, even able to reduce the salt content of saline wastewaters (Patel et al., 2021). There could be several different physiological mechanisms in the background of salinity tolerance in algae. These physiological mechanisms could be the operation of efficient Na^+^/K^+^ pump system (Asadian et al., 2018); ion accumulation (especially Na^+^ and Cl^−^) in newly formed or already existing vacuoles (Singh et al., 2018; Sahle-Demessie et al., 2019); and/or enhanced osmolyte production (Holzinger and Karsten, 2013). Salinity tolerance is not necessarily connected to salt (ion) accumulation: e.g., a salt tolerant *Dunaliella* strain was able to maintain intracellular Na^+^ concentrations lower than that of the environment (Pick et al., 1986). So, in addition to salt-tolerant or halophilic species, it is also worth taking into account the potential of freshwater green algae. It was proved that salt adapted strains could be generated from freshwater (salt sensitive) progenitors by selecting survivors of a progressively increasing salt stress (Perrineau et al., 2014; Li et al., 2018; Shetty et al., 2019). It was also proved that freshwater algae species can also provide isolates characterized by noteworthy salt tolerance and salt and nutrient removal ability (Figler et al., 2019, 2021). Next to “real” aquatic species (members of phytoplankton or phytobenthon), species from ephemeral aquatic habitats or aeroterrestrial algae also could be good candidates of research studying salt tolerance or desalination ability, since salinity stress is often referred as “physiological drought” (Kirst, 1990). Although, salinity stress and drought are different types of water unavailability, the acclimation responses to them could be comparable, since both affect the internal osmotic potential (Karsten, 2012). The main fundamental difference between the two phenomena is the changes of ion ratios (Holzinger and Karsten, 2013). During salt stress the ion ratio changes because of selective ion uptake (or release). In contrast, during drying out the ion ratios more or less remain the same beside the increasing ionic concentration (Holzinger and Karsten, 2013). The question arises, whether isolates form ephemeral aquatic habitats endangered by regular drying out (considered as special salt stress), could have higher salt tolerance and consequently better applicability in desalination processes than freshwater ones.

*Haematococcus lacustris* (Girod-Chantrans) Rostafinski is a flagellated green alga widely known and studied for its capacity to accumulate astaxanthin, a red keto-carotenoid pigment. The pigment has many applications in healthcare, cosmetic and food industries (Generalić Mekinić et al., 2023) and the natural-based astaxanthin is commercially produced by several companies (Mussagy et al., 2023). The green alga species was previously referred as *H. pluvialis*, but *H. lacustris* was regarded as the correct name of the type species (Nakada and Ota, 2016). The green alga is cosmopolitan, referred as a freshwater species (Guiry and Guiry, 2023), but actually the main habitats of the species are small, shallow ephemeral water bodies (bird baths, ornamental wells, other small natural or artificial pools; Genitsaris et al., 2016). However, the ionic composition and concentration of these habitats generally really fall into the range of freshwaters. These habitats can be characterized by many abiotic stress conditions, mainly by high extent of irradiation and regular, usually fast drying out. Survival of *H. lacustris* in such rapidly changing environment is ensured by its complex life cycle. Under unfavorable environmental conditions, the flagellated vegetative cells lose their flagella, and enter the so called palmella state (immobile vegetative cells). These cells can transform into asexual aplanospores over time under constant environmental stress (Shah et al., 2016). Aplanospores have a thick and rigid envelope and a resistant cell wall, making the cells able to withstand extreme environmental conditions (Shah et al., 2016) and enable the species to spread by zoochory or wind (Figuerola and Green, 2002; Genitsaris et al., 2011).

As it was mentioned above, algae isolates form ephemeral aquatic habitats endangered by regular drying out (exposed to special salt stress), could have notable salt tolerance and consequently successful applicability in desalination processes. The accumulation of valuable metabolites (such as astaxanthin) as a response to salinity stress, could enhance the economic value of the biomass. Therefore, in this study we aimed to investigate the salt and drying out tolerance, and the desalination and nutrient removal ability of the isolated *H. lacustris* strain. We hypothesized that increase in salt concentration by added salt (sodium chloride) would have a stronger effect on growth, cyst formation and carotenoid accumulation than volume decrease due to evaporation (drying out), since the model organism is not adapted to high chloride concentrations, but common in intermittent habitats from the freshwater range.

## 2 Materials and methods

### 2.1 Strain, culturing conditions, and experimental design

The strain used in the present work (ACCDH-UD1205) was isolated from an irrigation water storage barrel in the settlement of Ebes, eastern Hungary. Based on ITS rDNA (ITS1-2.8S-ITS2) sequences, it was found to be identical with NIES 144 and CCAP 34/1D strains and was identified as *H. pluvialis* (Allewaert et al., 2015). The isolate is maintained in the Algal Culture Collection of the Department of Hydrobiology, University of Debrecen in Optimized Haematococcus Medium (OHM; Fábregas et al., 2000) at 24°C under a photoperiod of 14 h of light (80 μmol photons m^−2^ s^−1^) and 10 h of darkness.

Sodium chloride treatments were carried out in sterile air bubbled cultures (with an approximate air flux 2.3 L min^−1^), in 250 mL Erlenmeyer flasks, with a final volume of 200 mL, among the above detailed circumstances. Salt concentrations: 100; 250; 500; 1,000; 2,000; 3,000, and 4,000 mg l^−1^ were used, which were adjusted by adding appropriate amounts of NaCl (VWR, Debrecen, Hungary) stock solution (300 g l^−1^). Control cultures did not contain added NaCl. Aeration was provided through glass tubes plugged into the mouth of the Erlenmeyer flasks by paper wool plugs. Paper wool allow liquid evaporation, so to avoid volume decrease and concomitant concentration increase and possible crystallization of the added salt, the volume of the cultures was maintained by the addition of sterile distilled water before every sampling events. The duration of the experiment was 11 days, because of the growth characteristics of the control culture among the applied circumstances (exponential phase till days 9–10).

Drying out treatments were also carried out in sterile air bubbled cultures, but in 100 mL Erlenmeyer flasks, with a final volume of 50 mL, among the above detailed circumstances. Lower culture volumes were used to reach significant volume decrease within reasonable time (modeling rapid drying out of a habitat). So called absolute control, control and drying out cultures were applied. In the case of the absolute control cultures, the amount of liquid lost as a result of sampling and evaporation was refilled with OHM to 50 mL at every sampling event (continuous nutrient replenishment for masking the physiological processes related to water shortage and nutrient deprivation). The volume replacement of the control culture was done by refilling with distilled water (no nutrient replenishment—only for masking the physiological processes related to water shortage). In the case of drying out cultures, the lost liquid was not replenished, as the purpose was to investigate the effects of drying out and the resulting increase in medium concentration. The duration of the experiment was 16 days to reach as much volume decrease as it was possible without the overaging and collapse of the control and absolute control cultures.

### 2.2 Culture growth measurements

The growth of the cultures was monitored by counting the number of cells. For counting, 200 μL samples were taken from the cultures on every second days and preserved with formaldehyde (5% final concentration; VWR, Debrecen, Hungary). Cell numbers were counted from 10 μL preserved samples in hemocytometer (Bürker chamber) using an Olympus BX50F-3 microscope (Olympus Optical Co., Ltd., Tokyo, Japan) at 400× magnification. Flagellated cells were referred as “vegetative cells,” cell types without flagella were referred as cyst during presentation of cell number changes. To give cell type proportions, the total cell number on the given sampling day was considered as 100%, and the proportions of certain cell types were given as percentages. Flagellated “green vegetative cells” (GV), carotenoid-accumulating flagellated cells as “green-red vegetative cells” (GRV), non-motile green cells as “green cysts” (GC), non-motile carotenoid-accumulating cells as “green-red cysts” (GRC) and matured aplanospores as “red cysts” (RC) were distinguished.

In order to calculate the NaCl concentrations that cause 50% growth inhibition (EC_50_ values), the degree of growth inhibition (in percentage, compared to the control) was plotted as a function of NaCl concentrations. Trend lines were fitted to the obtained curves, then the concentrations causing 50% inhibition were calculated using the equations of the trend lines (quadratic equations).

### 2.3 Conductivity, chloride, and nutrient content (nitrate and phosphate) measurements

Cultures were centrifuged at day 11 (salt treatment) and at day 16 (drying out) (10,000 rpm, 10 min, 24°C; Multifuge X4R Pro, Thermo Electron LED GmbH, Osterode am Harz, Germany). The pellets were freeze-dried (Christ Alpha 1–2 LD plus, Osterode, Germany) and stored at −20°C until further processing (pigment analysis). The conductivity, chloride, nitrate, and phosphate contents were measured from the cell-free supernatants.

Conductivity was measured with a Hach Lange HQ30d portable multimeter (Hach Lange GmbH, Düsseldorf, Germany) using an IntellicalTM CDC401 conductivity measuring electrode (Hach Lange GmbH, Düsseldorf, Germany). The extent of the decrease in conductivity to the given day (day 11 or 16) was given as percentage, the initial values were considered 100%.

To evaluate the desalination ability of the cells, the chloride content of the culturing medium was measured by precipitation titration (Németh, 1998). The amount of chloride was calculated using the formula provided by the method. The extent of chloride removal by the given day (day 11 or 16) was given as percentage, the initial values were considered as 100%.

Nitrate and phosphate contents were measured using spectrophotometric methods. In the case of nitrate, the salicylic acid - colorimetric method was used (Hungarian Standard MSZ 1484-13:2009, 2009). The acidic phosphorus-molybdenate method (Hungarian Standard MSZ EN ISO 6878:2004, 2004) was used to measure the phosphate content. The volume of both methods was reduced to minimize sample requirements, so the measurements were performed in Eppendorf tubes. The extent of nitrate and phosphate removal to certain days (day 11 or 16) was given as percentage, the initial values were considered 100%.

### 2.4 Analysis of pigment composition

Pigment contents of the collected biomasses were extracted with dimethyl sulfoxide (DMSO; Technical Report TR.1002.001, 1999). Briefly: 0.025 g aliquots of freeze-dried biomasses were extracted with 1 mL DMSO for 30 min with stirring (Velp Scientifica ESP, Usmate Velate, Italy) at 24°C in darkness. Each sample was then transferred into 1.5 mL Eppendorf tubes and centrifuged (16.2 × g, 5 min, Heraeus Fresco 17, Hanau, Germany), the supernatants were removed. The pellets were resuspended in 1 mL fresh DMSO, and the extraction was repeated, altogether 4 extraction cycles were implemented. The extraction was done in dim light circumstances to avoid degradation of the pigments (Ahmed et al., 2015). The removed supernatants of the 4 cycles were collected and stored in Packard cuvettes at −20°C in darkness.

Chlorophyll-a, -b and total carotenoid contents were measured spectrophotometrically from the DMSO extracts (Sumanta et al., 2014). The main carotenoid components (lutein/zeaxanthin, astaxanthin-esters and β-carotene) were separated and their amounts per dry weight were estimated by thin layer chromatography (TLC). For the TLC analysis, pigments were transferred from DMSO to ethyl-acetate according to Du et al. (2016) with modifications (Bácsi et al., 2018). Aliquots of 20 μL of the DMSO-free ethyl-acetate phases were subjected to TLC plates (Silica gel 60 GF254 10 × 20 cm; Merck, Darmstadt, Germany). For standard stock solution preparation, 1.0 mg from standard pigments (lutein, astaxanthin-esters mixture and β-carotene, Merck, Darmstadt, Germany) were dissolved in 2.0 mL DMSO. From all three stock solutions, 50 μL were measured to a 1.5 mL Eppendorf tube and the mixture was complemented to 400 μL with DMSO. The standards then were transferred from the DMSO solvent to ethyl-acetate similarly as the samples, and aliquots of 2.5; 5.0; 7.5, and 10.0 μL of the DMSO-free ethyl-acetate phases were subjected to the TLC plates. The standard solution and the samples were subjected to the TLC plates as bands according to the recommendations of Hynstova et al. (2018) with minor modifications: number of tracks was 12, band length was 5.0 mm, first horizontal application position was 15.0 mm (distance from each side), vertical application position was 20.0 mm (distance from lower edge). The running phase was petroleum ether:isopropanol:water 100:10:0.25 v/v (Hynstova et al., 2018), solvent front was 60.0 mm. After the run, the plates were dried at room temperature (5 min) and illuminated on the stage glass of a 3 M 9700 overhead projector (3 M Visual System Division, Austin, Texas, United States), photographed using a Nikon D3200 digital camera with AF-S DX NIKKOR 18–105 mm VR objective (Nikon Corporation, Tokio, Japan), and the images were analyzed with the GelAnalyzer 23.1 software (Lázár and Lázár, n.d.^1^).

### 2.5 Statistical analysis

All experiments were done in triplicates. Levene’s test was used to test the homogeneity of variances and Shapiro–Wilk test was used to check for normality.

The differences in the growth of the cultures over time, as well as the cell number and cell type ratio between the different treatments on each sampling day were evaluated by one-way analysis of variance (ANOVA).

To present the relationships between cell types and experimental treatments, the cell type proportion data (% of the total cell number on the given sampling day) of salt treatments as well as drying out experiments were subjected to principal component analysis (PCA).

The extent of conductivity reduction (%), chloride and nutrient removal (%), as well as the pigment composition changes were compared by one-way analysis of variance (ANOVA).

To show whether the conductivity, chloride content and nutrient content have changed significantly from the beginning to the end of the experiments, the results of the zero and the last day were compared with paired *t*-test. Also paired *t*-test were used to compare certain treatments of salt addition experiment and certain days of drying out experiment characterized by similar salinity. For statistical analyses, the PAST 4.13 software was used (Hammer et al., 2001).

## 3 Results

### 3.1 Growth and salt tolerance

**Figure 1:**
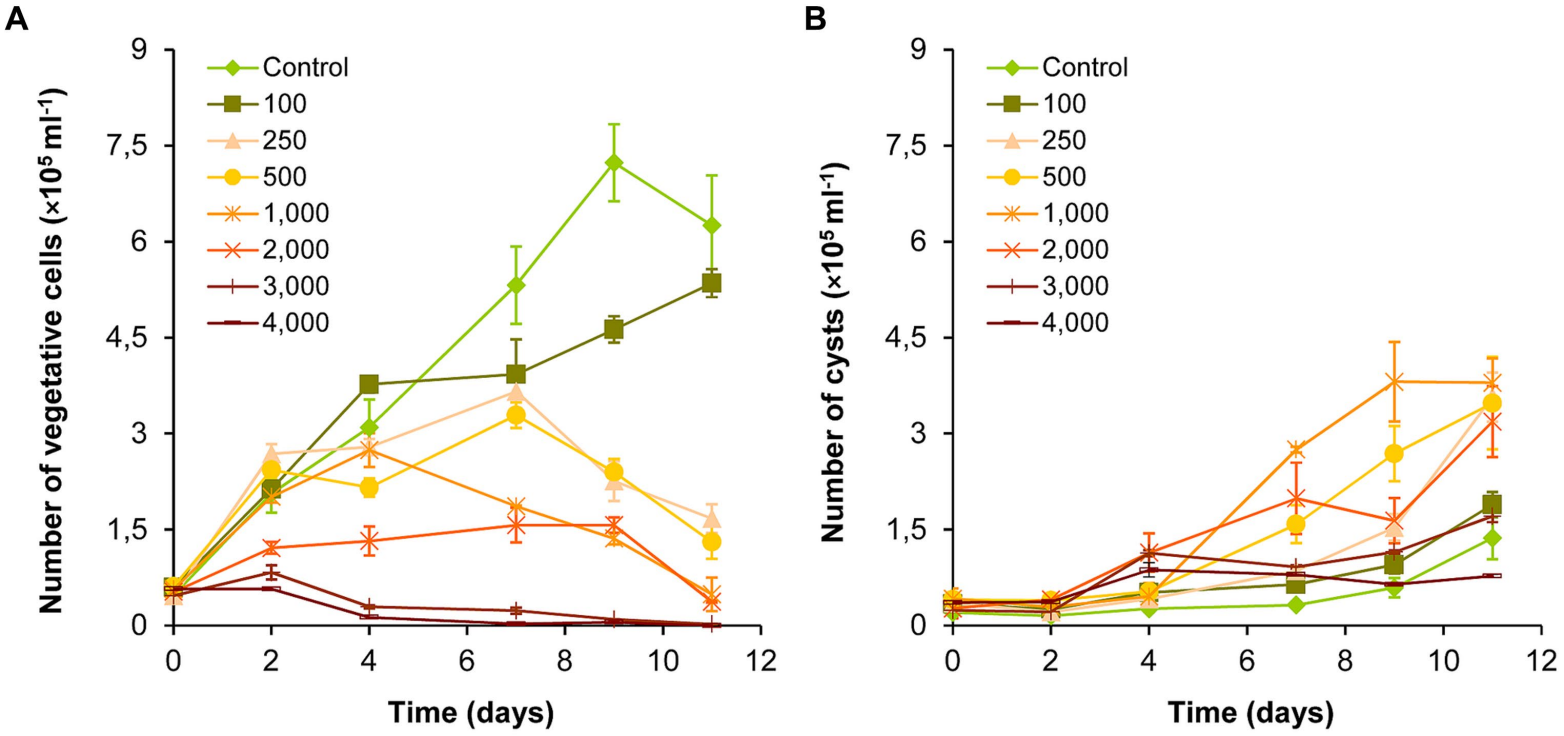
Changes in the numbers of vegetative cells **(A)** and cysts **(B)** in control and NaCl-treated *Haematococcus lacustris* cultures. The numbers (100–4,000) are the NaCl concentrations used in the treatments in mg l^−1^. The mean values and standard deviations are plotted (*n* = 3). The results of the statistical evaluation are summarized in Supplementary Table S1 (*p* < 0.05; ANOVA).

The number of vegetative cells in the control and 100 mg l^−1^ NaCl-treated cultures increased significantly over the entire exposure period (*p* < 0.05; Figure 1A; Supplementary Table S1A). There was also a significant increase in the 250 and 500 mg l^−1^ treatments until day 7 (*p* < 0.05), and then the number of vegetative cells decreased significantly until the end of the experiment (day 11; *p* < 0.05). The number of vegetative cells decreased after day 4 in the 1,000–2,000 mg l^−1^ NaCl treatments, and from day 2 in the 3,000–4,000 mg l^−1^ treatments (Figure 1A). Significant growth inhibition was observed in the cultures treated with 2,000–4,000 mg l^−1^ NaCl compared to the control and to the treatments 100–1,000 mg l^−1^ for the entire exposition time (*p* < 0.05). The 3,000 and 4,000 mg l^−1^ treatments did not differ significantly in the number of vegetative cells, which almost completely disappeared from these cultures by day 11 (Figure 1A; Supplementary Table S1A).

The number of cysts increased significantly from day 9 in control and in 100 mg l^−1^ treatment, or from day 7 in 250–1,000 mg l^−1^ treatments (*p* < 0.05; Figure 1B; Supplementary Table S1B). The number of cysts increased also in 2,000–4,000 mg l^−1^ treatments, but to a lesser extent with the increasing salt concentration (Figure 1B). Comparing the different treatments, the results show that the number of cysts increased in the concentration range of 100–1,000 mg l^−1^ NaCl in parallel with the increase of the salt concentration. Although, the number of cysts was higher in the treatments above 1,000 mg l^−1^ than in the control, significant differences could be detected only in a few cases (Supplementary Table S1B).

Regarding vegetative cell number, our results show that lower salt concentration was required over time to achieve 50% growth inhibition (Table 1). The concentration of NaCl causing 50% growth inhibition decreased substantially from day 4 to day 11. EC_50_ values calculated on the basis of total cell number, were higher than calculated for vegetative cells, but the trend (decrease from day 4 to day 11) was similar.

**Table 1:** NaCl concentrations causing 50% growth inhibition in NaCl treated *Haematococcus lacustris* cultures.

Cell types	EC_50_ (mg l^−1^ NaCl)		
	Day 4	Day 7	Day 11
Vegetative cells	1404.3 ± 46.9	937 ± 22	221.3 ± 3.1
Total number of cells	2852.5 ± 109.6	2048.3 ± 65.8	1389.5 ± 168.6

### 3.2 Growth and tolerance of drying out

**Figure 2:**
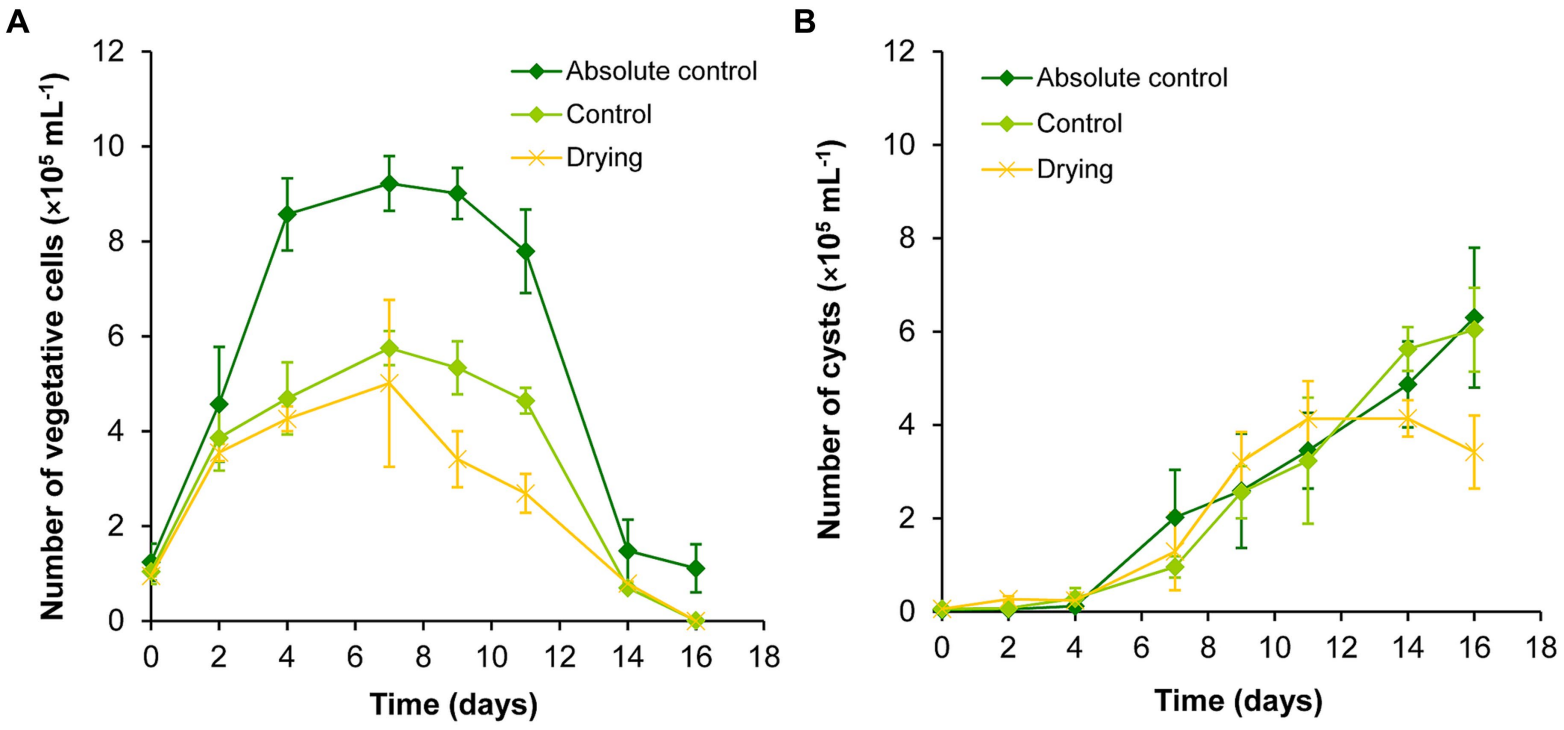
Changes in the numbers of vegetative cells **(A)** and cysts **(B)** in absolute control, control, and drying out *Haematococcus lacustris* cultures. The mean values and standard deviations are plotted (*n* = 3). The results of the statistical evaluation are summarized in Supplementary Table S2 (*p* < 0.05; ANOVA).

The volume of the absolute control and control cultures were successfully maintained during the drying out experiment (Supplementary Table S4). The volume of the drying out culture continuously decreased, it was significantly lower than the volume of controls from the 4th day of the experiment. The initial volume decreased by 75% to day 16 (Supplementary Table S4). Vegetative cells reached their highest number on day 7 in all cultures during the drying out experiment. In the absolute control culture, the vegetative cell number remained significantly higher on days 4–11 than on the first 2 days of the experiment. There were significantly less vegetative cells on days 14–16 than on previous days in all cultures (*p* < 0.05; Figure 2A; Supplementary Table S2A).

The number of cysts increased continuously in absolute control and control cultures throughout the whole experiment, there were significantly higher cyst number counts from day 7 (Figure 2B; Supplementary Table S2B). Cyst number also increased in drying out cultures to day 11, after that it decreased to the end of the experiment (Figure 2B). There were significantly higher cyst number counts in these cultures from day 9 than on the previous days, but cyst numbers did not change significantly from day 9 to day 16 (Supplementary Table S2B).

Comparing the different treatments, growth trends were similar in absolute control and control cultures, but there were significantly higher vegetative cell numbers in absolute control culture from day 4 (Figure 2; Supplementary Table S2). There were significantly lower vegetative cell numbers on day 9 and 11 in drying out cultures than in control (*p* < 0.05; Supplementary Table S2). There were no significant differences in the cyst numbers of the cultures (Supplementary Table S2).

Despite the similar growth trends of the control cultures of the two sets of experiments (salt treatment and drying out) from the start to day 11, there were notable differences. Vegetative cell numbers from the start to day 4 were significantly higher in control of drying out experiment than in control of salt treatment (*p* < 0.05). In the contrary, significantly higher vegetative cell numbers were detectable in control of salt treatment on days 9–11 (*p* < 0.05). Cyst numbers were significantly higher from day 7 in control of drying out experiment than in control of salt treatment (*p* < 0.05).

### 3.3 Cell type proportions

**Figure 3:**
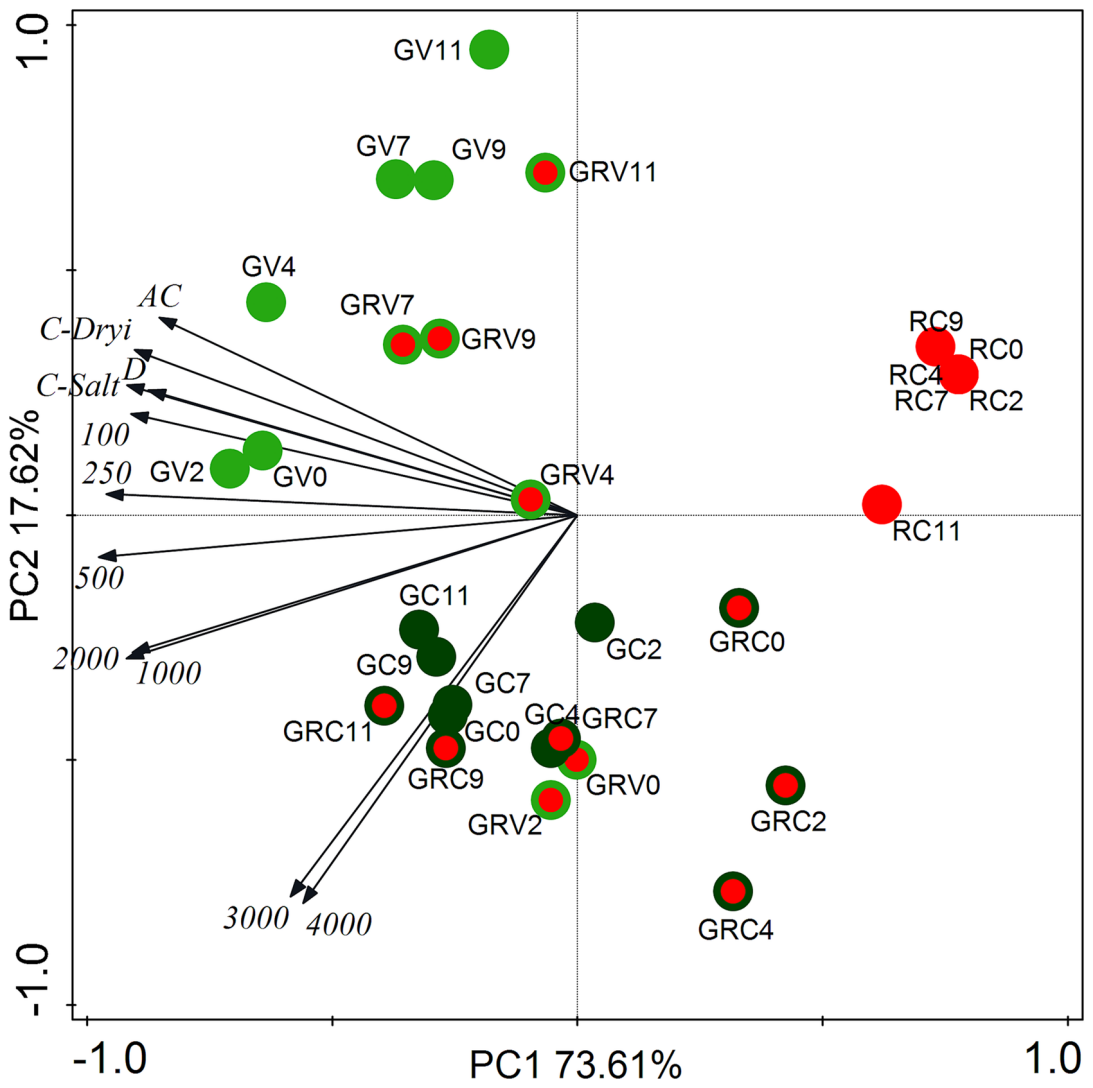
Score plot of the first two principal components from PCA of cell type proportion data from salt (NaCl) treatment dataset and from drying out experiment dataset. The numbers (100–4,000) are the NaCl concentrations used in the treatments in mg l^−1^. AC, absolute control; C, control; D, drying out; GV, green vegetative cells; GRV, green-red vegetative cells; GC, green cysts (palmella stage); GRC, green-red cysts; RC, red-cysts (matured aplanospores). The numbers 0–11 are the sampling days.

Cell type proportion data (% of the total cell number on the given sampling day) of salt treatments as well as drying out experiments were subjected to principal component analysis (PCA, Figure 3). The two axes explained 91.23% of the variances (Figure 3).

Proportions of green vegetative cells (GV) showed positive correlation with controls, lower salt concentration treatments (100–500 mg l^−1^) and drying out culture, especially in the first 4 days (GV0-4; Figure 3). The correlation of GV7-11 was weak, because of the decreasing proportion of GV on days 7–11 in all treatments (data not shown). It is worth to mention that proportions of GV started to decrease already from day 4 in the case of the highest salt concentrations (3,000 and 4,000 mg l^−1^; data not shown). Proportions of green-red vegetative cells (GRV) also correlated positively rather with controls, lower salt concentration treatments and drying out culture (Figure 3). Positive correlation of GRV0 and GRV2 with the highest salt concentrations was because of the high GRV proportions on days 0 and 2 in these cultures (data not shown). Proportions of green cysts (GC; actually the palmella stage) correlated positively with higher salt concentrations (1,000–4,000 mg l^−1^; Figure 3). Proportions of green-red cysts (GRC) showed positive correlation with the highest salt concentrations (3,000 and 4,000 mg l^−1^), especially GRC7-11 (Figure 3), since their proportions were the highest in these treatments on days 7–11 (data not shown). There was no correlation of red cysts (fully matured aplanospores) with the treatments, because they appeared only in cultures treated with 3,000 and 4,000 mg l^−1^ NaCl on day 11; or in drying out experiment after day 9 in low proportions (<5%; data not shown). Although, it should be mentioned that proportion of red cysts exceeded 20% in the drying out culture on days 14 and 16 (which were not included in the PCA analysis).

### 3.4 Conductivity reduction and chloride removal

**Figure 4:**
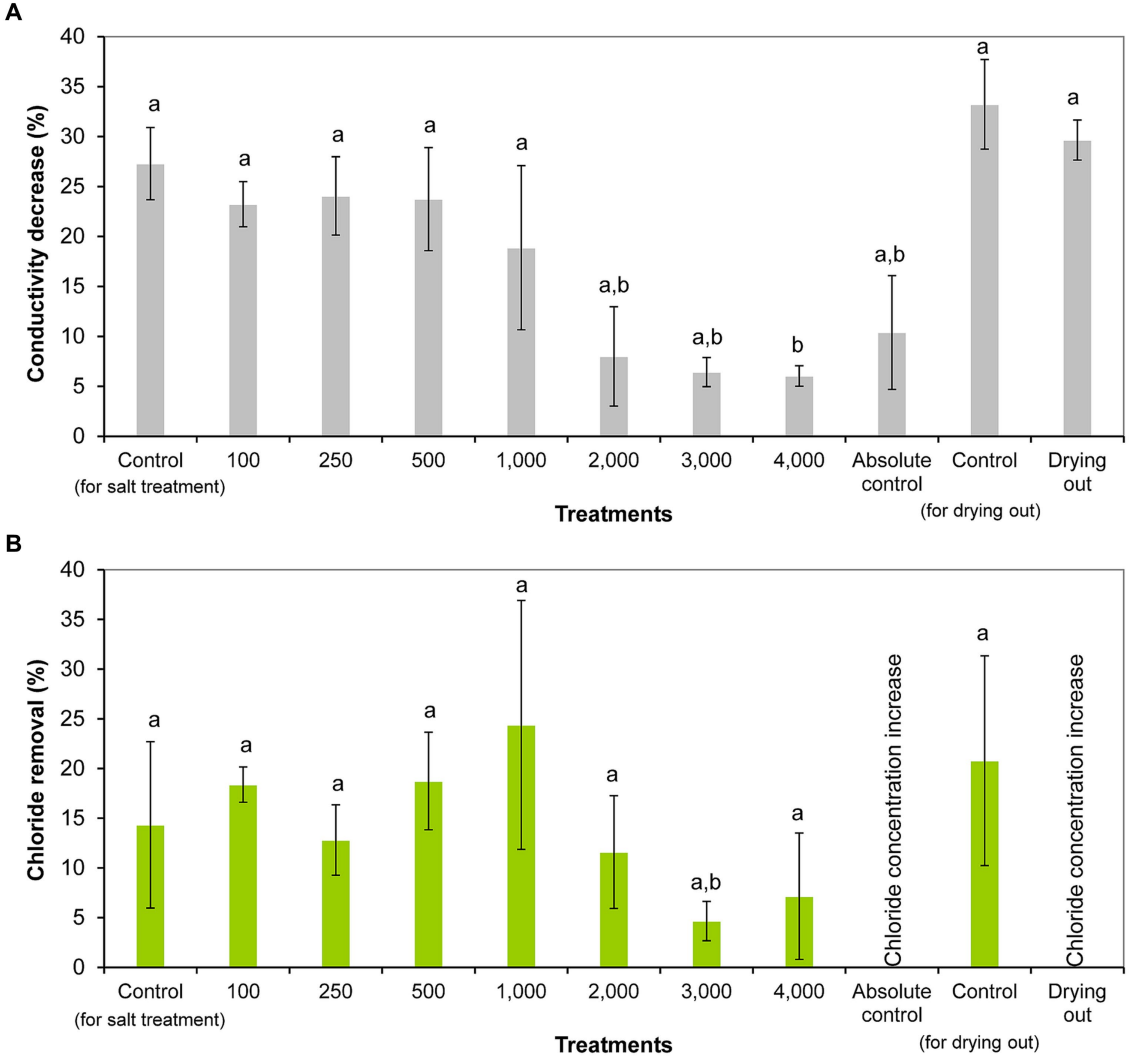
Conductivity reduction **(A)** and chloride removal **(B)** within 11 days of exposure given in percentages in the differently treated *Haematococcus lacustris* cultures. The numbers (100–4,000) are the NaCl concentrations used in the treatments in mg l^−1^. The mean values and standard deviations are plotted (*n* = 3). Different lowercase letters indicate significant differences (*p* < 0.05; ANOVA).

Conductivity was significantly reduced from day 0 to day 11 in all treatments (including controls) except 1,000 and 2,000 mg l^−1^ NaCl treatments and absolute control (*p* < 0.05; Supplementary Table S3). The decrease in conductivity ranged from 6% (4,000 mg l^−1^ treatment) to 27% (control) in salt treatment experiment, and from 10% (absolute control) to 33% (control) in the drying out experiment. It has to be emphasized that conductivity decreased also in drying out culture despite its significantly decreasing volume throughout the experiment (*p* < 0.05; Supplementary Table S4). Significantly less decrease in conductivity was observed in the cultures treated with 2,000–4,000 mg l^−1^ NaCl and in absolute control compared to the controls, treatments with lower NaCl concentrations, and drying out culture (*p* < 0.05; Figure 4A).

Chloride concentration decreased significantly in cultures treated with 100, 250, and 500 mg l^−1^ NaCl from day 0 to day 11 (*p* < 0.05; Supplementary Table S3). Chloride removal ranged from 4% (3,000 mg l^−1^ treatment) to 24% (1,000 mg l^−1^ treatment) in NaCl treated cultures (Figure 4B). It was observed that the degree of chloride removal decreased at higher salt concentrations (2,000–4,000 mg l^−1^), but there was no significant difference between the treatments (Figure 4B). It has to be noted that there was no chloride removal in absolute control and drying out cultures, their chloride concentrations increased significantly to day 11, and in the drying out culture further to day 16 (*p* < 0.05; Supplementary Table S3).

Comparing the chloride concentrations of the different settings, it should be noted that the chloride concentration of the drying out cultures was significantly higher on days 11 and 16 compared to the control and absolute control cultures (*p* < 0.01). Chloride concentration of the drying out culture on day 11 was comparable with the chloride concentration of 100 mg l^−1^ NaCl treatment on day 11. Chloride concentration of the drying out culture at the end of the experiment (on day 16) was comparable with the chloride concentration of 250 mg l^−1^ NaCl treatment on day 11 (Supplementary Table S3).

### 3.5 Nitrate and phosphate removal

**Figure 5:**
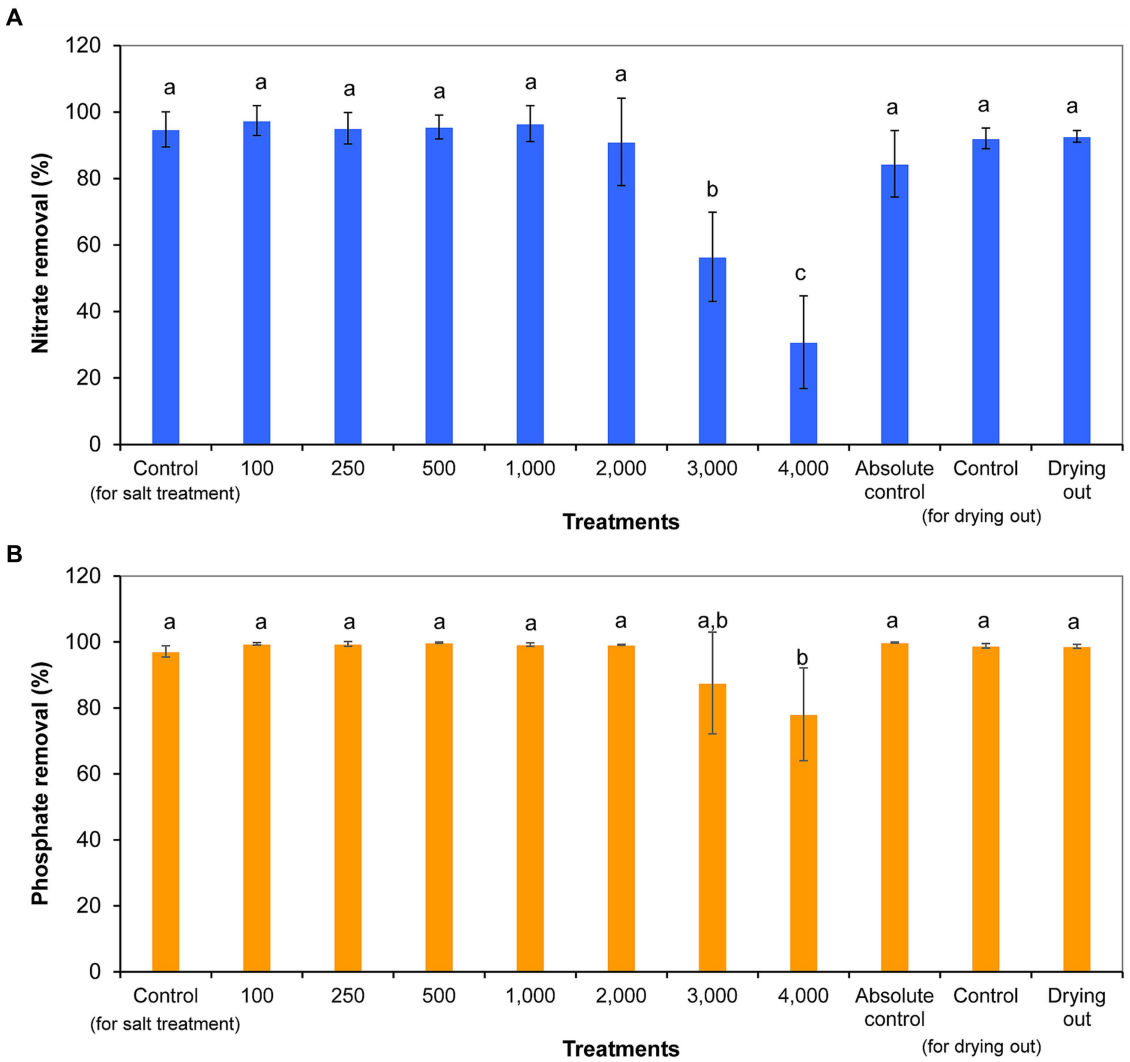
Nitrate **(A)** and phosphate **(B)** removal within 11 days of exposure given in percentages in differently treated *Haematococcus lacustris* cultures. The numbers (100–4,000) are the NaCl concentrations used in the treatments in mg l^−1^. The mean values and standard deviations are plotted (*n* = 3). Different lowercase letters indicate significant differences (*p* < 0.05; ANOVA).

Nitrate concentration decreased significantly from day 0 to day 11, with the exception of 4,000 mg l^−1^ NaCl treatment. Nitrate concentration did not show further significant decreases from day 11 to day 16 in the cultures of the drying out experiment (*p* < 0.05; Supplementary Table S5). Nitrate removal ranged from 31% (4,000 mg l^−1^ NaCl treatment) to 97% (100 mg l^−1^ NaCl treatment). It has to be noted that nitrate removal was over 80% even in absolute control culture, in which nutrient contents were continuously refreshed by volume maintenance with the addition of culturing medium. Cultures treated with 3,000 and 4,000 mg l^−1^ NaCl removed significantly less nitrate compared to control cultures and other treatments (Figure 5A).

Phosphate concentration decreased significantly from day 0 to day 11 in all cultures (*p* < 0.05; Supplementary Table S5). Phosphate concentration did not show further significant decreases from day 11 to day 16 in the cultures of the drying out experiment (*p* < 0.05; Supplementary Table S5). Phosphate removal ranged from 78% (4,000 mg l^−1^ NaCl treatment) to 100% (500 mg l^−1^ NaCl treatment and absolute control) in the cultures. The ~100% phosphate removal of absolute control culture is remarkable taking into account the continuous reload with the addition of culturing medium for volume maintenance. Phosphate removal was significantly lower in culture treated with 4,000 mg L^−1^ NaCl compared to control and other treated cultures (*p* < 0.05; Figure 5B).

### 3.6 Pigment content and composition

**Figure 6:**
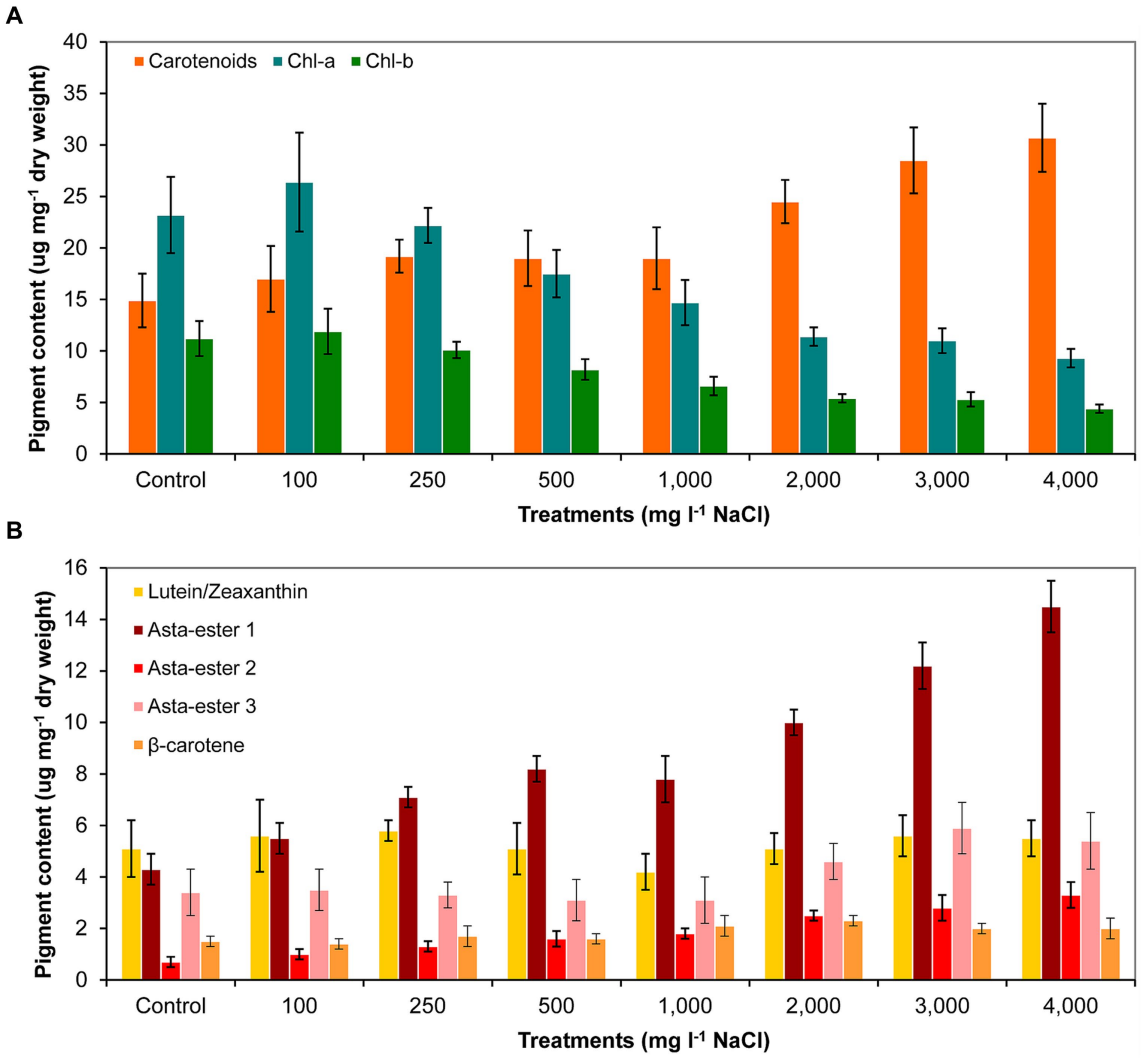
Pigment composition in control and NaCl-treated *Haematococcus lacustris* cultures at the end of the experiment (day 11) based on **(A)** spectrophotometric and **(B)** thin layer chromatographic analyses. The numbers (100–4,000) are the NaCl concentrations used in the treatments in mg l^−1^. Chl-a and -b: chlorophyll-a and -b; Asta-ester 1–3: the identified astaxanthin-esters. The mean values and standard deviations are plotted (*n* = 3). The results of the statistical evaluation are summarized in Supplementary Table S6 (*p* < 0.05; ANOVA).

The analysis of the pigment content supports the observations of the morphological changes. The carotenoid content indeed increased as a result of the salt treatment. However, it should be noted that the carotenoid content was significantly higher compared to control only from 2,000 mg l^−1^ and higher NaCl concentrations. In the cases of 3,000 and 4,000 mg l^−1^ NaCl treatments, significantly higher carotenoid content was also detectable compared to cultures treated with lower salt concentrations (*p* < 0.05; Figure 6A; Supplementary Table S6A). Chlorophyll-a and -b contents decreased with increasing salt concentration, significantly lower concentrations were measured from 1,000 mg l^−1^ NaCl than in the control culture (*p* < 0.05; Figure 6A; Supplementary Table S6A). Thin-layer chromatography analysis showed that the treatment with NaCl did not cause a change in the qualitative composition of the main identifiable carotenoids: neither new pigments were detected compared to control, nor did any metabolites disappear from those detectable in control (Supplementary Figure S1). However, clear quantitative changes occurred for the pigments identified as astaxanthin esters: their amounts increased with increasing salt concentration. Asta-ester 1 and 2 were present in significantly larger quantities from 1,000 mg l^−1^ NaCl concentration than in the control (*p* < 0.05; Figure 6B; Supplementary Table S6B). It is worth highlighting that at concentrations of 1,000–4,000 mg l^−1^ NaCl, the total amount of astaxanthin esters reached 1.3–2.3% of the dry weight. In the case of lutein/zeaxanthin, no clear trend was observed; the β-carotene content increased slightly with increasing salt concentration, but this change was not significant (Figure 6B; Supplementary Table S6B).

**Figure 7:**
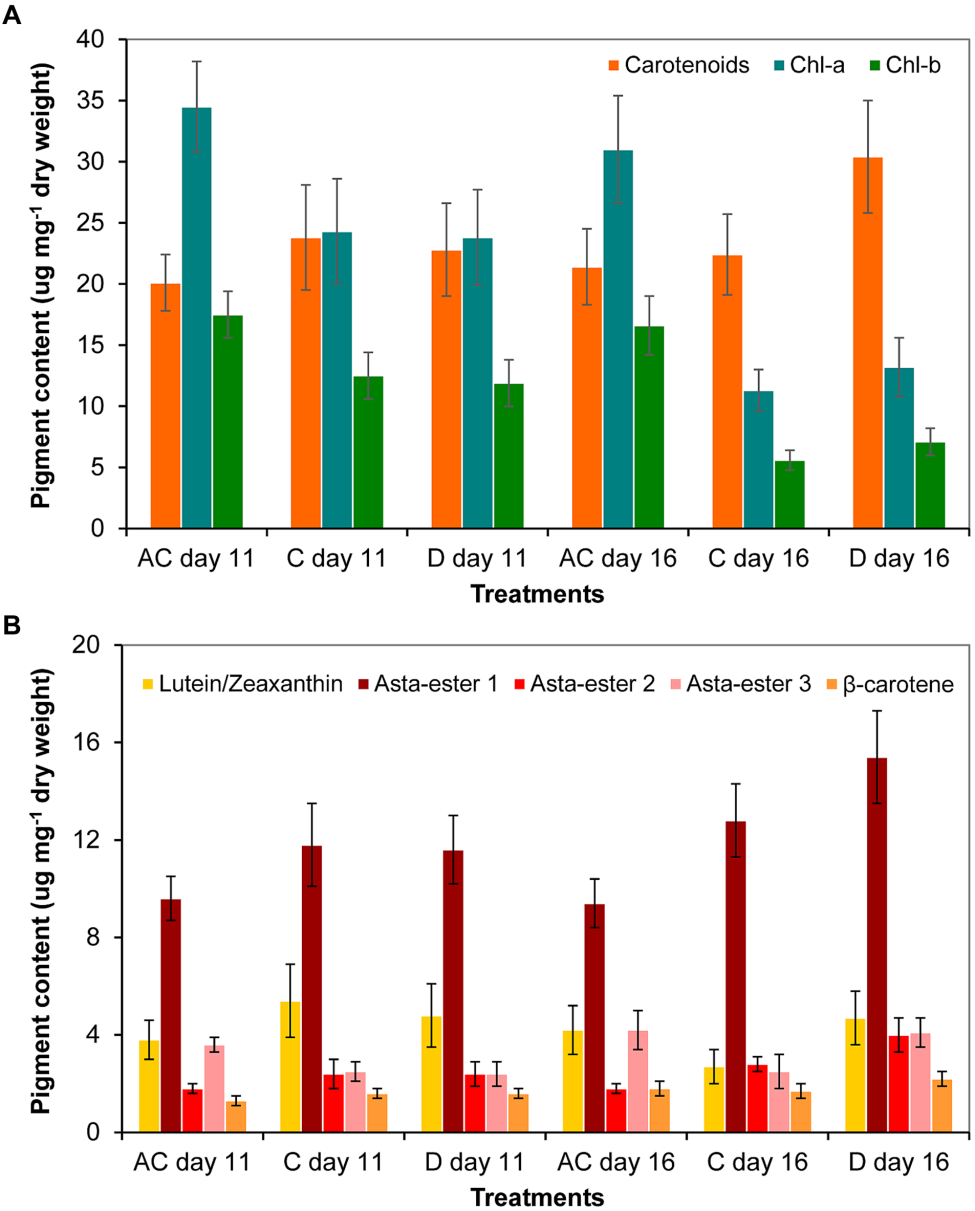
Pigment composition in absolute control (AC), control (C) and drying out (D) *Haematococcus lacustris* cultures on day 11 and 16 based on **(A)** spectrophotometric and **(B)** thin layer chromatographic analyses. Chl-a and -b: chlorophyll-a and -b; Asta-ester 1–3: the identified astaxanthin-esters. The mean values and standard deviations are plotted (*n* = 3). The results of the statistical evaluation are summarized in Supplementary Table S7 (*p* < 0.05; ANOVA).

As a result of the volume decrease (during drying out), the amount of carotenoids increased significantly only to day 16 in the drying out culture compared to control culture. The chlorophyll-a and -b contents in the absolute control culture were significantly higher on days 11 and 16 than in control and drying out cultures. The amount of chlorophylls in all cultures decreased significantly from day 11 to day 16 (*p* < 0.05; Figure 7A; Supplementary Table S6A). The thin-layer chromatography analysis showed that drying out did not cause any change in the qualitative composition of the main identifiable carotenoids: neither new pigments were detected compared to control, nor did any metabolites disappear from those detectable in control (Supplementary Figure S2). There was no significant change in the amount of identified carotenoids on day 11, the amount of Asta-ester 2 and 3 increased significantly in the drying out culture to day 16 (*p* < 0.05; Figure 7B; Supplementary Table S7B).

Comparing the two sets of experiments (salt treatment and drying out), the amount of carotenoids was significantly higher in control for drying out experiment than in control for salt treatment. In the case of cultures comparable on the basis of chloride content (see Supplementary Table S3), the carotenoid content was also higher on day 11 of drying out culture than on the day 11 of the 100 mg l^−1^ NaCl treatment. The 16-day-old drying out culture had significantly higher carotenoid and lower chlorophyll content than in the 11-day-old 250 mg l^−1^ NaCl treatment. Regarding the main identified carotenoids, the amount of Asta-ester 1 and 2 were significantly higher in the cultures of drying out experiment than in the corresponding cultures of salt treatment. The amount of β-carotene was also significantly higher in the drying out culture on day 16 than in the 250 mg l^−1^ NaCl treatment on day 11.

## 4 Discussion

### 4.1 Growth of the cultures

Based on cell number changes, the studied *H. lacustris* strain can be characterized by lower salt tolerance than other freshwater green algae investigated by our workgroup (Figler et al., 2019, 2021) or as green algae studied in other laboratories (Alvensleben et al., 2016). The vegetative cells showed low salt tolerance, survival could be ensured by the formation of cysts up to 2,000 mg l^−1^ NaCl concentration. For other *Haematococcus* isolates, there are relatively few data in the literature detailing the relationship between growth and salt concentration. Studies agree that salt concentrations of 0.8% (8,000 mg l^−1^) or higher cause complete inhibition of growth (Boussiba and Vonshak, 1991; Zharova et al., 2022). Chekanov et al. (2014) isolated a strain on Kost’yan island from a habitat characterized with extreme light, temperature and salinity changes. The strain can be considered as salt tolerant, able to grow under 25‰ (25,000 mg l^−1^) NaCl concentration. Three strains isolated in Australia by Gao et al. (2015) were also found to be salt tolerant [there was a detectable growth even at a concentration of 0.17 M (~10,000 mg l^−1^) NaCl]. The salt tolerance of *H. lacustris* used in this study can be considered as “average”: It showed growth even at 2,000 mg l^−1^ NaCl concentration (similar to other strains; Li et al., 2022; Zharova et al., 2022), but 4,000 mg l^−1^ NaCl has already inhibited growth, similarly to other observations (Li et al., 2022).

Surprisingly, the growth of the absolute control culture maintained at a constant volume with the OHM medium showed a similar trend to that observed in the control and drying out cultures. The essential difference was that the number of vegetative cells in this culture was significantly higher than in the other cultures, and almost the same amount of this large number of vegetative cells turned to cysts as in the other two cultures. This means that some of the vegetative cells died instead of turning into a cyst. One reason for the lower cyst formation observed in the absolute control culture could be the continuous nutrient replenishment, the another reason could be the shading effect due to the high cell density. A common phenomenon in *H. lacustris* strains is that cyst formation is most affected by the amount and quality of light (Borowitzka, 1992; Kobayashi et al., 1997; Oslan et al., 2021). Shading caused by high cell density may have prevented the conversion of vegetative cells to a higher proportion of cysts. Cyst formation observed in the control culture maintained at constant volume with sterile distilled water was most likely due to depletion of nutrients (nitrogen and phosphorus).

Although tolerance of dessication is already studied in the case of aeroterrestrial or aquatic green algae (Holzinger and Karsten, 2013; Pichrtová et al., 2014), even in the case of *Haematococcus* strains (Roach et al., 2022a,b), according to our knowledge there is much less information in the literature about the growth of algae in habitats with decreasing volume (i.e., during the process of drying out). It seems that growth characteristics of the studied strain were not remarkably affected by drying out under the applied circumstances, only from day 9 (Figure 2A; Supplementary Table S2A), when volume loss was around 40% (Supplementary Table S4). The reason for the lower number of cysts in the drying out culture from day 11 could be the significantly lower vegetative cell number from day 7; fewer cysts could be formed from the fewer vegetative cells by the end of the experiment.

In conclusion, it can be said, that the hypotheses about stronger effect of NaCl addition on growth than drying out, was proved. The background of this could be that the species adapted more to even fast drying out than to drastic increase of Na^+^ and Cl^−^ ion concentrations (ephemeral habitats generally with low salinity; Genitsaris et al., 2016; Guiry and Guiry, 2023). This assumption is also supported by chloride removal ability (see below in chapter 4.3).

Growth differences between the control cultures of the two sets of experiments (salt treatment and drying out) can certainly be explained by the different volumes (habitat size). The size (volume) of the habitat and the volume-to-surface ratio could be important features from the point of view of algal growth (Marino et al., 2011; Nayana et al., 2022). In the salt treatment and drying out experiments the volume-to-surface ratio was significantly different (1:0.16 and 1:0.6, respectively). The salt treatments took place in a larger volume (to avoid crystallization in the case of higher concentrations), while lower volume was applied for the drying out experiment to reach significant volume reduction within a reasonable time. The light conditions of the culture in the larger volumes of NaCl treatments were certainly significantly different from those in the smaller volumes of drying out experiments. During drying out, the layer thickness of the culturing medium continuously decreased, and the surface area increased (due to the shape of the Erlenmeyer flask). As a result of the decrease in volume, due to the smaller light path, a much higher light intensity could reach the cells, contributing to the observed differences not just in growth, but in cell types and pigment contents as well (see below).

### 4.2 Cell type proportions

Most of the literature demonstrated that motile cells lose their flagella with increasing salinity, pigment accumulation occurs in the emerging non-motile cells (Boussiba and Vonshak, 1991; Cordero et al., 1996; Gao et al., 2015; Liu et al., 2021; Zharova et al., 2022). The dynamics of the process, as well as the salt concentration at which the individual morphological changes occur, show a great variety between the different strains. The loss of flagella was reported already after 24 h with 0.1% (~1,000 mg l^−1^) and 86 mM (~5,000 mg l^−1^) NaCl (Cordero et al., 1996; Liu et al., 2021). The appearance of the red pigment content was observed after 1–2 days (Gao et al., 2015), the majority of cells transformed into red aplanospores in 3–5 days (Zharova et al., 2022). In the case of the strain investigated in the present study, a more significant difference from general observations is that completely red cysts appeared late (after 9 days at the earliest) and in a relatively small proportion. The reason for this can certainly be the lower light intensity used in this study than reported in the literature. The other difference is that proportion of the motile cells accumulating carotenoids (GRV) reached a ratio of up to 60% by the 7th day of the experiment (100 mg l^−1^ salt treatment; absolute control). Based on literature data, pigment accumulation in motile cells is not particularly characteristic, although the phenomenon is known in the case of some isolates (Grünewald et al., 1997; Hagen et al., 2001; Brinda et al., 2004; Del Río et al., 2005, 2008; García-Malea et al., 2009; Tocquin et al., 2012; Butler et al., 2017). According to the latest observations, carotenoid accumulation started in the motile cells under moderate light intensity (Li et al., 2018, 2022). Based on the results for the strain studied in the present work, pigment synthesis was induced in the motile vegetative cells under conditions of moderate stress, i.e., salt stress that has not yet caused vegetative cell death, presumed nutrient deficiency, and moderate light intensity. Further investigation of the phenomenon would be definitely justified, due to the advantageous properties of motile vegetative cells compared to cysts from a biotechnological point of view (Butler et al., 2017; Li et al., 2022).

As a final conclusion, it can be said that the hypothesis about stronger/sooner induction of cyst formation by addition of NaCl than drying out, was proved.

### 4.3 Conductivity and chloride content changes

There are only a few data in the literature, how freshwater algae are able to reduce conductivity or chloride content of their environment. Our results show that the extent of conductivity reduction caused by the *H. lacustris* strain is similar to that of *Chlorococcum* sp. (Figler et al., 2019) and *Coelastrum morus* (Figler et al., 2021) isolates. In the case of the 1,000 mg l^−1^ NaCl treatment, it was lower than the values measured in the cultures of the other isolates (Figler et al., 2019). Conductivity decreased also in drying out culture despite its significantly decreasing volume throughout the experiment. Complex ion-exchange processes could be the background of the phenomenon, the explanation of them is beyond the scope of this study, but it can be concluded that conductivity measurement is not necessarily suitable to detect concentration process parallel to volume decrease.

The decrease of chloride content of the *H. lacustris* cultures showed a similar pattern to that of *Desmodesmus spinosus* and *Monoraphidium pusillum* strains, and its extent was similar to that of the *Desmodesmus communis* isolate (Figler et al., 2019). Based on the results, the studied *H. lacustris* strain is able to reduce the chloride content of the medium, however, this is not particularly significant and decreases at higher salt concentrations (2,000–4,000 mg l^−1^ NaCl). Since the survival is ensured by cyst formation, probably ion accumulation by the vegetative cells as protective mechanisms against salt stress is not characteristic to *H. lacustris* above certain concentration. However, it should be emphasized, that a ~180 mg l^−1^ chloride concentration decrease was observed in the 1,000 mg l^−1^ NaCl treated culture (Supplementary Table S3), which could be considered as significant, since domestic and industrial wastewater could contain this or higher amount of chloride ions (Cao et al., 2022). Chloride content of absolute control and drying out cultures increased to day 11 (and to day 16 in the latter case). In absolute control, the continuous addition of the culturing medium explains this observation. The increasing chloride concentration of the drying out culture is a proof for concentration processes parallel to the decreasing volume of the culture. Since chloride is not accumulated extensively, the remaining amount is present in gradually decreasing volume, resulting increase in concentration.

### 4.4 Nutrient removal

The results show that nutrient removal was not directly affected by salt concentration, only indirectly through growth inhibition. It is worth to mention that the level of phosphate removal was significant even in the presence of high NaCl concentrations, probably thanks to the high nitrogen:phosphorus ratio (6 N:1P) of the culturing medium (Shriwastav et al., 2014; Choi and Lee, 2015; Liu and Vyverman, 2015; Alketife et al., 2017; Arora et al., 2019).

Nutrient uptake is strongly species, or even strain dependent. The studied *H. lacustris* strain was able to take up high amounts of nitrate and phosphate. The extent of removal was over 80% for nitrate and nearly 100% for phosphate despite the continuous replacement of nutrients by addition of culturing medium in the case of absolute control culture. This highly intensive nutrient uptake ability can probably be explained by the characteristics of the natural habitat of the species. *H. lacustris* strains generally appear among nutrient poor environment (Genitsaris et al., 2011, 2016).

### 4.5 Pigment composition changes

In the light of the literature data, it can be said that the pigment composition of the studied strain changed in a general way. After a different time and to a different extent, but in all of the reported isolates the chlorophyll content decreased and the carotenoid content increased as a result of NaCl treatment (Boussiba and Vonshak, 1991; Cordero et al., 1996; Li et al., 2022). The astaxanthin content of the strain investigated in this study can be considered as average: 8.34 mg g^−1^ total astaxanthin (0.8% of dry weight) was detected in the control culture on day 11. As a result of NaCl addition, this amount increased to 17.14 mg g^−1^ (1.7% of dry weight) on day 11 in the culture treated with 2,000 mg l^−1^ NaCl, in which detectable vegetative cell proliferation still occurred. During an experiment with a similar duration, Li et al. (2022) achieved 25.92 mg g^−1^ astaxanthin content in cultures treated with the same salt concentration, although it should be noted that they used a significantly higher light intensity (250 μmol m^−2^ s^−1^) than the one was used in the present study (80 μmol m^−2^ s^−1^). Literature data suggest that in the case of isolates more sensitive to salinity, the highest astaxanthin content can be achieved at lower salt concentrations: e.g. in the case of the strain studied by Li et al. (2022), the astaxanthin content was lower at 4,000 mg l^−1^ NaCl (17.26 mg g^−1^) than at 2,000 mg l^−1^ NaCl (25.92 mg g^−1^), and than in the strain investigated in the present study also at 4,000 mg l^−1^ NaCl (23.2 mg g^−1^). At the same time, the accumulation of pigments, including astaxanthin, of the more salt-tolerant strains can be induced by using a higher salt concentration (0.17 M; 10,000 mg/L, Gao et al., 2015) or cannot be induced solely by increasing the salt content. The strain isolated by Chekanov et al. (2014) did not accumulate astaxanthin at 25‰ (25,000 mg l^−1^) salt, astaxanthin accumulation was inducible only by additional nutrient deficiency and intensive illumination (Chekanov et al., 2014).

The results of the drying out experiment are difficult to compare with literature data, because according to our knowledge, there are no examples with similar experimental setup. In the work of Roach et al. (2022a,b) the cells were exposed to slow dessication with the complete lack of culturing medium (liquid phase), while in the experiment presented in this work, the cells were in the liquid phase throughout the whole experimental time, although with a constant volume decrease. In the experiment of Roach et al. (2022a,b), the astaxanthin content was significantly higher from the 4th day than in the control culture, and continued to increase until the 7th day due to dehydration. In our case, only the astaxanthin content of the 16-day-old drying out culture was significantly higher than that of the control. Thus, the time required for the induction of carotenoid synthesis during drying out appears to depend on the rate of the process.

During the two sets of experiments in this study (salt treatment and drying out), there were differences between the pigment contents of the controls and between the cultures characterized by similar chloride contents. The assumption, according to which salt addition has a stronger effect than volume decrease due to evaporation (drying out), was justified: morphological and carotenoid content changes occurred sooner during salt treatment than during drying out. Still, the differences are obviously partly caused by the different culture volumes and the concomitant differences in light regimes of the cultures (see above). The 11-day-old cultures were clearly deficient in nutrients, but based on these data, it is not possible to say how soon a significant nutrient deficiency was developed in the cultures. However, it can be assumed with certainty that in addition to salinity and volume changes, depletion of nutrients contributes to pigment composition changes (Boussiba and Vonshak, 1991; Boussiba et al., 1992; Fábregas et al., 2003; He et al., 2007; Kang et al., 2007; Yin et al., 2007; Meng et al., 2009).

## 5 Conclusion

In summary, the *H. lacustris* strain isolated from ephemeral habitat in eastern Hungary can be characterized by a general moderate salinity tolerance characteristic to other strains of the species, although vegetative growth can be expected up to 2,000 mg l^−1^ NaCl concentration. The strain is able to significantly reduce the conductivity of the culturing medium in the presence of NaCl up to 500 mg l^−1^. Chloride removal is not particularly characteristic to the strain, on the other hand, it can be characterized by an outstanding nitrate and phosphate removal capacity. The carotenoid content (including astaxanthin) of the strain can be considered as average, but it can be effectively induced, up to a level above 2% of dry weight, which is generally considered significant in the literature. The induction based not only on increasing salinity but the developing nutrient deficiency and changing light circumstances also contribute.

In conclusion, the strain could be at least as potent candidate of further bioremediation and related valuable biomass production research as other strains of the species, or other green algae from ephemeral aquatic habitats. Carotenoid accumulating ability in the motile phase is also worth further investigation. Finally, since the strain showed similar trends in cyst formation and pigment accumulation as other strains of the species, it can be regarded that *H. lacustris* is an excellent model organism for resting stage formation and related metabolite accumulation studies not just from biotechnological, but also from ecological point of view.

## Data availability statement

The original contributions presented in the study are included in the article/Supplementary material, further inquiries can be directed to the corresponding author.

## Author contributions

IB: Writing – review & editing, Writing – original draft, Visualization, Validation, Supervision, Resources, Project administration, Methodology, Funding acquisition, Formal analysis, Conceptualization. AF: Writing – review & editing, Writing – original draft, Visualization, Methodology, Investigation, Formal analysis, Conceptualization. ES: Writing – review & editing, Methodology, Formal analysis. MY: Writing – review & editing, Investigation, Formal analysis. KM: Writing – review & editing, Visualization, Investigation, Formal analysis. VB-B: Writing – review & editing, Writing – original draft, Visualization, Methodology, Formal analysis, Conceptualization.

## Footnotes

^1^ http://www.gelanalyzer.com
