# Supplementary material for: Salinity tolerance and desalination properties of a Haematococcus lacustris strain from eastern Hungary
Source: Front Microbiol. 2024 Mar 14;15:1332642. doi: 10.3389/fmicb.2024.1332642 (PMC10977603; doi:10.3389/fmicb.2024.1332642)
Supplement: Supplementary file 1 [file Table_1.pdf]

Table S1. Data of cell numbers (means±SD; n=3) in control and NaCl treated (100 – 4,000 mg l^{-1}) *Haematococcus lacustris* cultures.

a) Number of vegetative cells (×10^5 ml^{-1})

	0	2	4	7	9	11
Control	0.5±0.1 a	2.1±0.3 b	3.1±0.4 c	5.3±0.6 d	7.2±0.6 e	6.3±0.8 f
100	0.6±0.0 a	2.1±0.1 b	3.8±0.1 c	3.9±0.5 c *	4.6±0.2 d *	5.4±0.2 e *
250	0.5±0.1 a	2.7±0.2 b	2.8±0.1 b	3.7±0.3 c *	2.3±0.3 d **	1.7±0.2 e **
500	0.6±0.1 a	2.4±0.1 b	2.2±0.1 c	3.3±0.2 d *	2.4±0.2 b **	1.3±0.3 e **
1,000	0.6±0.0 a	1.8±0.4 b	2.4±0.8 b	1.9±0.2 b **	1.5±0.2 a **	0.5±0.2 a **
2,000	0.5±0.1 a	1.2±0.1 b *	1.3±0.2b *	1.6±0.1 b.c **	1.6±0.1 b.c **	0.4±0.0 a **
3,000	0.5±0.1 a	0.8±0.1 b *	0.3±0.0 c *	0.2±0.0 c ***	0.1±0.0 d ***	n.d.
4,000	0.6±0.1 a	0.6±0.0 a *	0.1±0.0 b *	0.0±0.0 c ***	0.1±0.0 b ***	n.d.

b) Number of cysts (×10^5 ml^{-1})

	0	2	4	7	9	11
Control	0.2±0.1 a	0.2±0.0 a	0.3±0.1 a	0.3±0.0 a	0.6±0.2 a.b	1.4±0.3 b
100	0.3±0.1 a	0.3±0.0 a	0.5±0.1 a.b	0.6±0.0 a.b	1.0±0.1 b	1.9±0.2 c
250	0.3±0.2 a	0.2±0.0 a	0.4±0.1 a	0.9±0.1 b	1.5±0.2 c	3.6±0.4 d *
500	0.4±0.1 a	0.4±0.0 a *	0.5±0.1 a	1.6±0.3 b *	2.7±0.4 c	3.5±0.7 d *
1,000	0.5±0.1 a	0.3±0.1 a	0.9±0.5 a	2.7±0.1 b **	3.4±1.3 b	3.9±0.2 b *
2,000	0.3±0.2 a	0.4±0.0 a *	1.1±0.3 b	2.0±0.6 c *	1.6±0.4 b.c	3.2±0.6 d *
3,000	0.2±0.0 a	0.2±0.0 a	1.1±0.0 b	0.9±0.0 c	1.1±0.0 b	1.7±0.1 d
4,000	0.4±0.1 a	0.4±0.1 a *	0.9±0.1 b	0.8±0.1 b	0.6±0.0 c	0.8±0.0 b

Different lowercase letters indicate significant differences between days (0 - 11) within the same experimental setup (rows; $p<0.05$; rm ANOVA).
Asterisks indicate significant differences between different treatments on the given days (columns; $p<0.05$; ANOVA).
n.d.: not detected
