# Supplementary material for: Salinity tolerance and desalination properties of a Haematococcus lacustris strain from eastern Hungary
Source: Front Microbiol. 2024 Mar 14;15:1332642. doi: 10.3389/fmicb.2024.1332642 (PMC10977603; doi:10.3389/fmicb.2024.1332642)
Supplement: Supplementary file 2 [file Table_2.pdf]

Table S2. Data of cell numbers (means±SD; n=3) in absolute control, control and drying out *Haematococcus lacustris* cultures.

a) Number of vegetative cells ($\times 10^5$ ml^{-1})

	0	2	4	7	9	11	14	16
Absolute control	1.2±0.4 a	4.6±1.2 b	8.6±0.8 c	9.2±0.6 c	9.0±0.5 c	7.8±0.9 d	1.5±0.7 a	1.1±0.5 a
Control	1.0±0.3 a	3.9±0.7 b	4.7±0.9 c *	5.8±0.4 d *	5.3±0.6 c *	4.6±0.3 b.c *	0.7 a	n.d.
Drying out	1.0±0.0 a	3.6±0.2 b	4.3±0.3 b *	5.0±1.8 b *	3.4±0.6 b **	2.7±0.4 b *	0.8 a	n.d.

b) Number of cysts ($\times 10^5$ ml^{-1})

	0	2	4	7	9	11	14	16
Absolute control	n.d.	0.1 a	0.1±0.1 a	2.0±1.0 b	2.6±1.2 b	3.5±0.8 b	4.9±0.9 c	6.3±1.5 c
Control	0.1±0.1 a	0.3±0.4 a	0.5±0.5 a	1.0±0.3 a	2.6±0.6 b	2.9±0.9 b	5.5±0.3 c	5.7±0.5 c
Drying out	0.1 a	0.3±0.1 a	0.2±0.2 a	0.3±0.8 b	3.2±0.6 c	4.1±0.8 c	4.1±0.4 c	3.4±0.8 c *

Different lowercase letters indicate significant differences between days (0 - 16) within the same experimental setup (rows; $p<0.05$; rm ANOVA).
Asterisks indicate significant differences between different treatments on the given days (columns; $p<0.05$; ANOVA).
n.d.: not detected
