# Supplementary material for: Salinity tolerance and desalination properties of a Haematococcus lacustris strain from eastern Hungary
Source: Front Microbiol. 2024 Mar 14;15:1332642. doi: 10.3389/fmicb.2024.1332642 (PMC10977603; doi:10.3389/fmicb.2024.1332642)
Supplement: Supplementary file 3 [file Table_3.pdf]

Table S3. Conductivity and chloride concentrations at the beginning (day 0) and at the end (day 11 and 16) of the salt treatment and drying out experiments (means±SD; n=3).

	Conductivity (µS cm^{-1})			Chloride (mg l^{-1})		
	day 0	day 11	day 16	day 0	day 11	day 16
Control (for salt)	915.7 ±22.9	665.7±32.9*	-	61.1±10.2	52.7±13.0	-
100	1088.3±39.5	835.0±13.5*	-	123.0±4.5	100.4±1.8*	-
250	1454.7±112.6	1103.0±68.2*	-	224.4±15.7	195.5±14.0*	-
500	2023.7±202.4	1536.3±49.6*	-	400.1±22.0	324.4±7.4*	-
1,000	2937.3±412.8	2390.0±95.4	-	671.2±177.1	492.8±53.7	-
2,000	4446.7±142.9	4086.7±116.8	-	1144.5±207.2	1005.2±127.4	-
3,000	6315.0±105.0	5910.0±190.0*	-	1522.9±20.7	1452.3 ±49.8	-
4,000	8070.0±208.8	7583.3±259.7*	-	2218.8±278.4	2053.3±186.7	-
Absolute control	935.0±60.4	835±11.0	715.0±28.4*	46.1±4.2	78.7±7.2*	89.1±5.0*
Control (for drying)	955.7±56.2	639.7±68.1*	271.7±66.5**	52.7±3.5	41.8±5.0*	40.2±0.2*
Drying out	962.0±30.0	676.7±23.6*	632.7±12.7*	49.7±1.9	116.3±12.6*	172.2±10.0**

The numbers (100 – 4,000) are the NaCl concentrations used in the treatments in mg l^{-1}.
Asterisks indicate significant differences between day 0 and day 11 or day 16 values (rows; p<0.05; paired t-test, ANOVA).

Table S4. Volume (ml) data in absolute control, control and drying out *Haematococcus lacustris* cultures (means±SD; n=3).

	0	2	4	7	9	11	14	16
Absolute control	47.0 ±1.0 a	46.3 ±1.5 a	47.0 ±4.0 a	46.7 ±1.5 a	48.3 ±1.5 a	47.3 ±2.5 a	48.7 ±1.5 a	45.7 ±1.5 a
Control	48.3 ±0.5 a	49.0 ±1.0 a	47.0 ±3.0 a	44.0 ±1.0 a	46.7 ±1.5 a	42.3 ±1.5 a	43.7 ±2.5 a	47.0 ±1.0 a
Drying out	48.5 ±0.5 a	43.7 ±0.6 a	38.5 ±0.5 b *	34.0 ±5.0 c *	28.7 ±4.5 d *	22.3 ±4.7 e *	15.7 ±3.7 f *	12.3 ±2.1 g *

Different lowercase letters indicate significant differences between days (0 - 16) within the same experimental setup (rows; p<0.05; rm ANOVA).
Asterisks indicate significant differences between different treatments on the given days (columns; p<0.05; ANOVA).
