# Supplementary material for: Salinity tolerance and desalination properties of a Haematococcus lacustris strain from eastern Hungary
Source: Front Microbiol. 2024 Mar 14;15:1332642. doi: 10.3389/fmicb.2024.1332642 (PMC10977603; doi:10.3389/fmicb.2024.1332642)
Supplement: Supplementary file 4 [file Table_4.pdf]

Table S4. Volume (ml) data in absolute control, control and drying out *Haematococcus lacustris* cultures (means±SD; n=3).

| | 0 | 2 | 4 | 7 | 9 | 11 | 14 | 16 |
|---|---|---|---|---|---|---|---|---|
| Absolute control | 47.0 ±1.0 a | 46.3 ±1.5 a | 47.0 ±4.0 a | 46.7 ±1.5 a | 48.3 ±1.5 a | 47.3 ±2.5 a | 48.7 ±1.5 a | 45.7 ±1.5 a |
| Control | 48.3 ±0.5 a | 49.0 ±1.0 a | 47.0 ±3.0 a | 44.0 ±1.0 a | 46.7 ±1.5 a | 42.3 ±1.5 a | 43.7 ±2.5 a | 47.0 ±1.0 a |
| Drying out | 48.5 ±0.5 a | 43.7 ±0.6 a | 38.5 ±0.5 b * | 34.0 ±5.0 c * | 28.7 ±4.5 d * | 22.3 ±4.7 e * | 15.7 ±3.7 f * | 12.3 ±2.1 g * |

Different lowercase letters indicate significant differences between days (0 - 16) within the same experimental setup (rows; $p<0.05$; rm ANOVA).

Asterisks indicate significant differences between different treatments on the given days (columns; $p<0.05$; ANOVA).
