# Supplementary material for: Salinity tolerance and desalination properties of a Haematococcus lacustris strain from eastern Hungary
Source: Front Microbiol. 2024 Mar 14;15:1332642. doi: 10.3389/fmicb.2024.1332642 (PMC10977603; doi:10.3389/fmicb.2024.1332642)
Supplement: Supplementary file 5 [file Table_5.pdf]

Table S5. Nitrate and phosphate concentrations at the beginning (day 0) and at the end (day 11 and 16) of the salt treatment and drying out experiments (means±SD; n=3).

	Nitrate (mg l^{-1})			Phosphate (mg l^{-1})		
	day 0	day 11	day 16	day 0	day 11	day 16
Control	262.0±52.7	13.2±13.2*	-	9.8±1.2	0.3±0.2*	-
100	176.2±89.0	6.8±11.8*	-	10.9±1.5	0.1±0.0*	-
250	240.0±176.8	9.6±14.5*	-	10.1±1.4	0.1±0.1*	-
500	203.4±65.0	9.3±8.5*	-	10.9±2.4	n.d.	-
1,000	272.9±70.9	8.8±12.8*	-	12.0±0.9	0.1±0.1*	-
2,000	183.8±6.4	17.1±25.2*	-	12.0±1.5	0.1±0.0*	-
3,000	246.5±21.6	109.1 ±43.1*	-	12.1±1.5	1.4 ±1.3*	-
4,000	201.8±15.1	138.8±22.7	-	11.6±1.3	2.6±1.7*	-
Absolute control	188.5±22.7	27.8±12.5*	17.3±6.3*	15.0±1.2	<0.1*	<0.1*
Control (for drying)	207.7±4.3	16.4±5.2*	25.8±1.0*	17.1±0.4	0.2±0.1*	0.1±0.1*
Drying out	238.1±49.2	16.7±0.5*	13.2±3.5*	16.0±3.6	0.2±0.1*	0.1±0.1**

The numbers (100 – 4,000) are the NaCl concentrations used in the treatments in mg l^{-1}.

Asterisks indicate significant differences between day 0 and day 11 or day 16 values (rows; p<0.05; paired t-test, ANOVA).
