# Supplementary material for: Salinity tolerance and desalination properties of a Haematococcus lacustris strain from eastern Hungary
Source: Front Microbiol. 2024 Mar 14;15:1332642. doi: 10.3389/fmicb.2024.1332642 (PMC10977603; doi:10.3389/fmicb.2024.1332642)
Supplement: Supplementary file 6 [file Table_6.pdf]

Table S6. Pigment content in control and NaCl treated (100 – 4,000 mg l^{-1}) *Haematococcus lacustris* cultures at the end of the experiment (day 11; means±SD; n=3).

a) Pigment content based on spectrophotometry (µg mg^{-1} dry weight)

	Carotenoids	Chlorophyll-a	Chlorophyll-b
Control	14.9±2.6 a	23.2±3.7 a	11.2±1.7 a
100	17.0±3.2 a	26.4±4.8 a	11.9±2.2 a
250	19.2±1.6 a	22.2±1.7 a	10.1±0.8 a
500	19.0±2.7 a	17.5±2.3 a,b	8.2±1.0 a,b
1,000	19.0±3.0 a	14.7±2.2 b	6.6±0.9 b
2,000	24.5±2.1 a,b	11.4±0.9 b	5.4±0.4 b
3,000	28.5±3.2 b	11.0±1.2 b	5.3±0.7 b
4,000	30.7±3.3 b	9.3±0.9 b,c	4.4±0.4 b,c

b) Estimated amounts of carotenoids identified by thin layer chromatography (µg mg^{-1} dry weight)

	Lutein/Zeaxanthin	Asta-ester 1	Asta-ester 2	Asta-ester 3	β-carotene
Control	5.1±1.1a	4.3±0.6 a	0.7±0.2 a	3.4±0.9 a	1.5±0.2 a
100	5.6±1.4 a	5.5±0.6 a	1.0±0.2 a	3.5±0.8 a	1.4±0.2 a
250	5.8±0.4 a	7.1±0.4 a,b	1.3±0.2 a	3.3±0.5 a	1.7±0.4 a
500	5.1±1.0 a	8.2±0.5 a,b	1.6±0.3 a	3.1±0.8 a	1.6±0.2 a
1,000	4.2±0.7 a	7.8±0.9 a,b	1.8±0.2 a,b	3.1±0.9 a	2.1±0.4 a
2,000	5.1±0.6 a	10.0±0.5 b	2.5±0.2 b	4.6±0.7 a	2.3±0.2 a,b
3,000	5.6±0.8 a	12.2±0.9 c	2.8±0.5 b	5.9±1.0 b	2.0±0.2 a
4,000	5.5±0.7 a	14.5±1.0 d	3.3±0.5 b	5.4±1.1 a	2.0±0.4 a

Different lowercase letters indicate significant differences between the different treatments ($p<0.05$; ANOVA).
