# Supplementary material for: Salinity tolerance and desalination properties of a Haematococcus lacustris strain from eastern Hungary
Source: Front Microbiol. 2024 Mar 14;15:1332642. doi: 10.3389/fmicb.2024.1332642 (PMC10977603; doi:10.3389/fmicb.2024.1332642)
Supplement: Supplementary file 7 [file Table_7.pdf]

Table S7. Pigment content in the *Haematococcus lacustris* cultures of the drying out experiment on day 11 and day 16 (means±SD; n=3).

a) Pigment content based on spectrophotometry (µg mg^{-1} dry weight)

		Carotenoids	Chlorophyll-a	Chlorophyll-b
	Absolute control	20.1±2.3 a	34.5±3.7 b	17.5±1.9 b
Day 11	Control	23.8±4.3 a	24.3±4.3 a	12.5±1.9 a
	Drying out	22.8±3.8 a	23.7±3.9 a	11.9±1.9 a
	Absolute control	21.4±3.1 a	31.0±4.4 b	16.6±2.4 b
Day 16	Control	22.4±3.3 a	11.3±1.7 b	5.6±0.8 b
	Drying out	30.4±4.6 b	13.2±2.4 b	7.1±1.1 a

b) Estimated amounts of carotenoids identified by thin layer chromatography (µg mg^{-1} dry weight)

		Lutein/Zeaxanthin	Asta-ester 1	Asta-ester 2	Asta-ester 3	β-carotene
	Absolute control	3.8±0.8 a	9.6±0.9 a	1.8±0.2 a	3.6±0.3 a	1.3±0.2 a
Day 11	Control	5.4±1.5 a	11.8±1.7 a	2.4±0.6 a	2.5±0.4 a	1.6±0.2 a
	Drying out	4.8±1.3 a	11.6±1.4 a,b	2.4±0.5 a	2.4±0.5 a	1.6±0.4 a
	Absolute control	4.2±1.0 a	9.4±1.0 a	1.8±0.2 a	4.2±0.8 a,b	1.7±0.3 a
Day 16	Control	2.7±0.7 a	12.8±1.5 a	2.8±0.3 a	2.5±0.7 a	1.7±0.3 a
	Drying out	4.7±1.1 a	15.4±1.9 a,b	4.0±0.7 b	4.1±0.6 b	2.2±0.3 a,b

Different lowercase letters indicate significant differences between the different treatments ($p<0.05$; ANOVA).
