# Supplementary material for: Salinity tolerance and desalination properties of a Haematococcus lacustris strain from eastern Hungary
Source: Front Microbiol. 2024 Mar 14;15:1332642. doi: 10.3389/fmicb.2024.1332642 (PMC10977603; doi:10.3389/fmicb.2024.1332642)
Supplement: Supplementary file 8 [file Image_1.pdf]

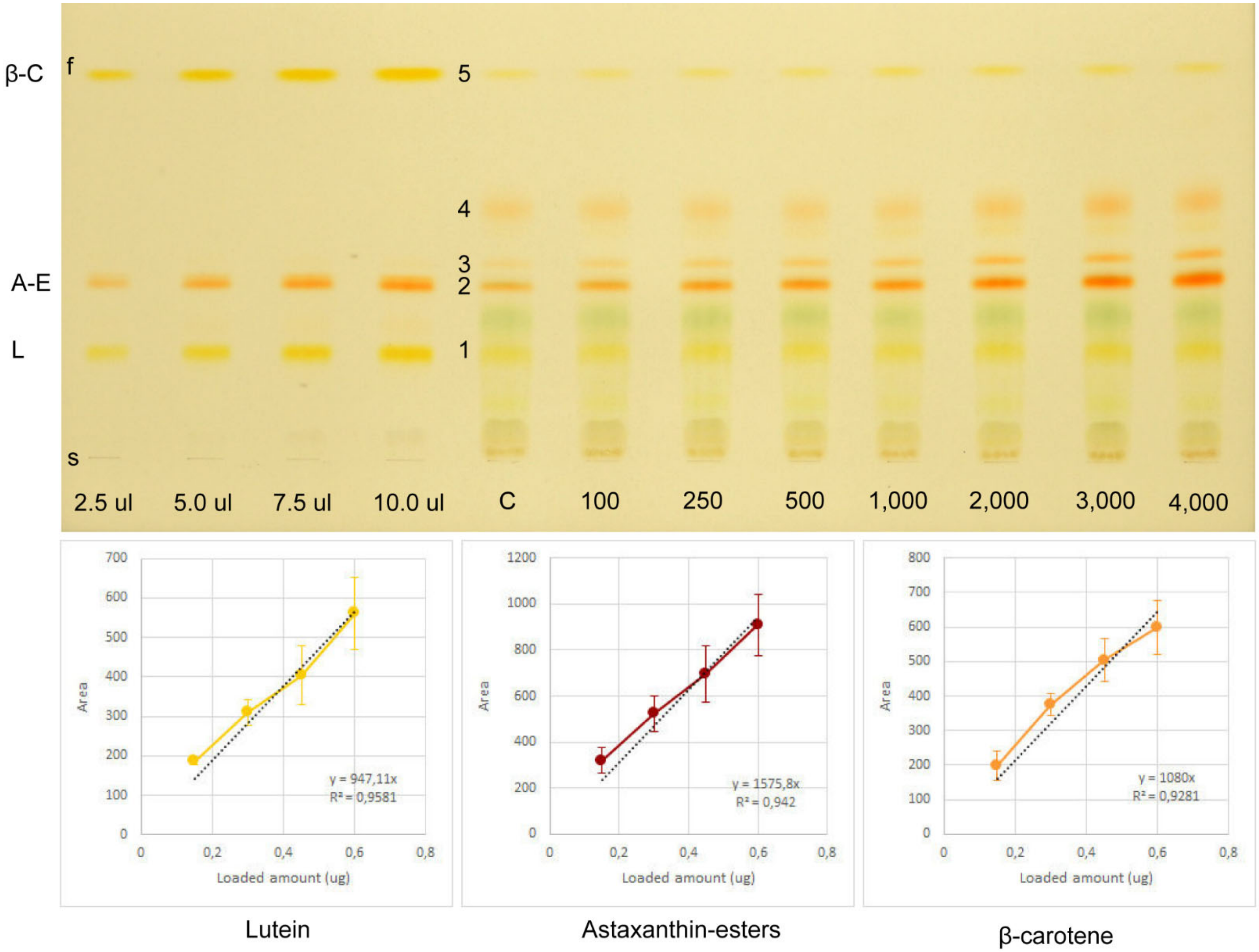

Figure S1 Thin layer chromatography of control and NaCl treated *Haematococcus lacustris cultures*. Standards: β-C: β-carotene; A-E: Astaxanthin-esters; L: lutein. 2.5 – 10.0 µl: loaded volumes of the standard mix solution. C: control; 100 – 4,000: NaCl concentrations in mg l^{-1}. The identified pigments; 1: lutein/zeaxanthin; 2 – 4: astaxanthin esters; 5: β-carotene. The graphs showing the standard curves for the corresponding pigment standards.
